# Supplementary material for: Identification of Freezing-Responsive microRNAs and Their Targets in Chinese Jujube by Small RNA and Degradome Sequencing
Source: Int J Mol Sci. 2026 Sep 4;27(17):7912. doi: 10.3390/ijms27177912 (PMC13566890; doi:10.3390/ijms27177912)
Supplement: Supplementary file 1 [file ijms-27-07912-s001.zip › Supplementary Material S2/novel_miR_32.pdf]

Provisional ID	:	novel_miR_32
Score total	:	1272.5
Score for star read(s)	:	3.9
Score for read counts	:	1266
Score for mfe	:	1
Score for randfold	:	1.6
Score for cons. seed	:	
Total read count	:	2495
Mature read count	:	2179
Loop read count	:	0
Star read count	:	316

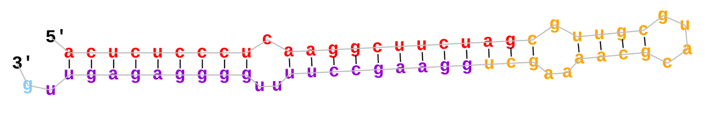

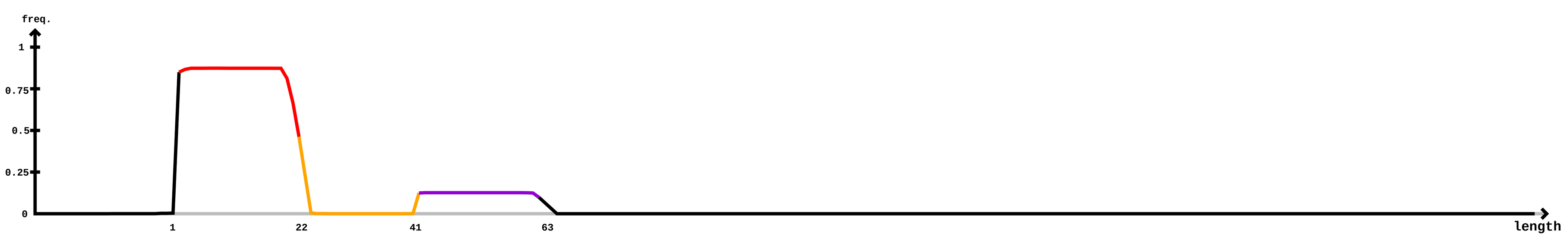

Mature Star

```
5'- uuauggugacaauuguuuguucacucucccucaaggcuucuagcguugcguacgcaaaagcuggaagccuuuuggggagaguugacagauuguugucacaccuuuuugcaguuuucuacuccuuggacuaguaauauuauuuuauauguaauaucacugcuuuacuuugccuacuugcacucucucuuaucauguuuccuaguuuccauuuuucugaaauuuuguugcaauuugugcugauuaugcuuac -3'  obs
    uuauggugacaauuguuuguucacucucccucaaggcuucuagcguugcguacgcaaaagcuggaagccuuuuggggagaguugacagauuguugucacaccuuuuugcaguuuucuacuccuuggacuaguaauauuauuuuauauguaauaucacugcuuuacuuugccuacuugcacucucucuuaucauguuuccuaguuuccauuuuucugaaauuuuguugcaauuugugcugauuaugcuuac        exp
    .....(((((((.((((((.(((((((((.(((((((((((.((((....))))..))))))))))))..))))))))).))))))).))))))))........((((..((((.....)))).((((.(((((((......))))))).))))........(((......)))............(((..(..((((((.........))))))..)..)))......))))..........       reads  mm  sample
    ...........auuguuuguucacucuccc...          1   0   LT-1
    ...................uucacucucccucaaggcu...  1   0   LT-1
    ...................uucacucucccucaaggcuuc...  1   0   JCK-3
    ...................uucacucucccucaaggcuuc...  3   0   JCK-2
    .....................cacucucccucaaggcuucu...  2   0   JCK-3
    .....................cacucucccucaaggcuucua...  1   0   LT-3
    ......................acucucccucaaggcuuc...  11  0   LT-1
    ......................acucucccucaaggcuuc...  3   0   JCK-3
    ......................acucucccucaaggcuuc...  14  0   LT-3
    ......................acucucccucaaggcuuc...  18  0   LCK-3
    ......................acucucccucaaggcuuc...  3   0   JCK-1
    ......................Ccucucccucaaggcuuc...  1   1   LCK-1
    ......................acucucccucaaggcuuc...  4   0   JCK-2
    ......................acucucccucaaggcuuA...  1   1   LT-3
    ......................acucucccucaaggcuuc...  40  0   LCK-2
    ......................acucucccucaaggcuuG...  1   1   JCK-1
    ......................acucucccucCaggcuuc...  1   1   LCK-2
    ......................acucucccucaaggcuuc...  19  0   LT-2
    ......................acucucccucaaggcuuc...  32  0   LCK-1
    ......................acucucccucaaggcuuUu...  3   1   JCK-2
    ......................acucucccucaaggcuucu...  34  0   JCK-3
    ......................acucucccucaaggcuucu...  57  0   LT-3
    ......................acucucccucaaggcuucu...  23  0   LT-1
    ......................acucucccucaaggcuuUu...  4   1   LT-3
    ......................aNucucccucaaggcuucu...  1   1   JCK-2
    ......................acucucccucCaggcuucu...  1   1   JCK-3
    ......................acucucccucaaggcuuUu...  1   1   JCK-1
    ......................acucucccucaaggcuuAu...  1   1   JCK-3
    ......................acucucccucCaggcuucu...  1   1   LCK-3
    ......................acucucccucaaggcuucu...  105 0   LCK-3
    ......................acucucccucaaggcuuUu...  1   1   JCK-3
    ......................acucucccucaaggcuucu...  1   0   JT-2
    ......................acucucccucaCggcuucu...  1   1   JCK-2
```

Mature Star

uuauggugacaauuguuuguucacucucccucaaggcuucuagcguugcguacgcaaaagcuggaagccuuuuggggagaguugacagauuguugucacaccuuuuugcaguuuucuacuccuuggacuaguaauauuauuuuauauguaauaucacugcuuuacuuugccuacuugcacucucucuuaucauguuuccuaguuuccauuuuucugaaauuuuguugcaauuugugcugauuaugcuuac

acucucccucaaggcuucA	2	1	LT-2
acucucccucaagAcuucu	1	1	LT-2
acucucccucaaggcuuAu	1	1	JCK-1
acucucccucaaggcuucu	52	0	JCK-2
acucucccucaaggcuucu	39	0	LT-2
acucucccucaaggcuucu	31	0	JCK-1
acucucccucaaggcuuAu	1	1	JCK-2
acucucccucaagAcuucu	1	1	LT-3
acucucccucaNggcuucua	1	1	JCK-1
acucucccucaaggcuucua	31	0	LT-1
acucucccucaaggcuucua	9	0	JT-2
acucucccucaaggcuucuC	1	1	LT-1
Ccucucccucaaggcuucua	1	1	JCK-1
acucucccucaaggcuucua	51	0	JCK-1
acucucccucaaggcuuUua	3	1	JCK-3
acucucccucCaggcuucua	1	1	LT-1
acucucccucaaggcuuUua	2	1	JCK-2
acucucccucaaggcuucua	30	0	JT-1
acucucccucaaggcuucua	33	0	LT-2
acucucccucaagAcuucua	1	1	JCK-1
acucucccucaaggcuucua	21	0	JT-3
acucucccucaaggcuucua	82	0	LCK-3
acucucccucaaggcuuUua	1	1	JCK-1
acucCcccucaaggcuucua	1	1	JCK-2
acucucccucaaggcuucuU	3	1	LT-2
acucucccucaaggcuucuU	8	1	JCK-2
acucucccucaaggcuucua	77	0	JCK-2
acucucccucaaggcuucua	46	0	LT-3
acucucccucaaggcuucua	58	0	JCK-3
acucucccucaaggcuucuU	6	1	JCK-1
acucucccucaaggcuucuU	3	1	JCK-3
acucucccucCaggcuucua	1	1	LT-3
acucucccucaaggcuucuag	160	0	JCK-3
acucucccucaaggcuucuaU	2	1	JCK-3
Ccucucccucaaggcuucuag	1	1	LT-2
acucucccucaaggcuucuNg	1	1	JCK-1
acucucccucaaggcuucuag	16	0	JT-3
acucucccucaaggcuucuaU	2	1	JCK-2
acucucccucaaggcuucuaA	3	1	JCK-2
acucuAccucaaggcuucuag	1	1	JCK-3
acucucccucaaggcuucuag	185	0	JCK-2
acucucccuUaaggcuucuag	1	1	JCK-3
Ccucucccucaaggcuucuag	1	1	JCK-3
acucucccucaaggcuucuag	71	0	LCK-3
acucucccuAaaggcuucuag	1	1	JCK-1
acucucccucaaggcuucuaA	1	1	JCK-1
acucucccucaCggcuucuag	1	1	JCK-2
acucucccucaCggcuucuag	1	1	LT-3
acucucccucaaggcuucuag	105	0	JCK-1
acucucccucaaggcuucuag	23	0	LT-2
acucucccucaaggcuucuag	14	0	LT-1
acucucccucaaggcuucuag	6	0	JT-2
acuGucccucaaggcuucuag	1	1	JCK-1
acucucccucaaggcuucuag	34	0	LT-3
acucucccucaaggcuucuagc	48	0	JCK-1
acucucccucaaggcuucuagU	36	1	JCK-3
acucucccucaaggcuucuagU	46	1	JCK-2
acucucccucaaggcuucuagU	21	1	LT-2
acucucccucaaggcuucuagU	9	1	LCK-3
acucucccucaaggcuucuagU	12	1	LT-1
acucucccucaaggcuucuagc	30	0	LT-3
acucucccucaaggcuucuagA	8	1	LCK-3
acucCcccucaaggcuucuagc	1	1	JCK-2
acucucccucaaggcuucuagA	7	1	JCK-1
acucucccucaaggcuucuagc	79	0	JCK-3
acucucccucaaggcuucuagc	21	0	LCK-3
acucucccucaaggcuucuagA	8	1	JCK-2
acucucccucaaggcuucuagU	21	1	LT-3
acucucccucaaggcuucuagU	28	1	JCK-1
acucucccucaaggcuucuagN	1	1	JCK-1

Mature Star

```
uuauggugacaauuguuuguucacucucccucaaggcuucuagcguugcguacgcaaaagcuggaagccuuuuggggagaguugacagauuguugucacaccuuuuugcaguuucuacuccuuggacuaguaauauuauuuuauauguaauaucacugcuuuacuuugccuacuugcacucucuuaucauguuuccuaguuuccauuuuucugaaauuuuguugcaauuugugcugauuaugcuuac
......................acucucccucaaggcuucuagA...........................................................................................................................................................................................    12    1    JCK-3
......................Ccucucccucaaggcuucuagc...........................................................................................................................................................................................    1     1    JCK-1
......................acucucccucaaggcuucuagc...........................................................................................................................................................................................    75    0    JCK-2
......................acucucccucaaggcuucuagG...........................................................................................................................................................................................    1     1    JCK-1
......................acucucccucaaggcuucuNgc...........................................................................................................................................................................................    1     1    JCK-1
......................acuGucccucaaggcuucuagc...........................................................................................................................................................................................    1     1    JCK-1
......................acucucccucaaggcuucuagc...........................................................................................................................................................................................    10    0    LT-1
......................Ccucucccucaaggcuucuagc...........................................................................................................................................................................................    1     1    JCK-3
......................acucucccucaaggcuucuagc...........................................................................................................................................................................................    7     0    JT-1
......................acucucccucaaggcuucuagc...........................................................................................................................................................................................    16    0    LT-2
......................acucucccucaaggcuucuagcg..........................................................................................................................................................................................    2     0    JCK-3
......................acucucccucaaggcuucuagcguu........................................................................................................................................................................................    1     0    LT-1
.......................cucucccucaaggcuucu..............................................................................................................................................................................................    2     0    JCK-3
.......................cucucccucaaggcuucu..............................................................................................................................................................................................    4     0    LT-3
.......................cucucccucaaggcuucua.............................................................................................................................................................................................    2     0    LT-3
.......................cucucccucaaggcuucua.............................................................................................................................................................................................    1     0    LT-2
.......................cucucccucaaggcuucua.............................................................................................................................................................................................    17    0    LCK-3
.......................cucucccucaaggcuucua.............................................................................................................................................................................................    4     0    JCK-2
.......................cucucccucaaggcuucuU.............................................................................................................................................................................................    1     1    LT-3
.......................cucucccucaaggcuucuU.............................................................................................................................................................................................    4     1    LCK-3
.......................cucucccucaaggcuucua.............................................................................................................................................................................................    1     0    LT-1
.......................cucucccucaaggcuucuag............................................................................................................................................................................................    2     0    JCK-1
.......................cucucccucaaggcuucuaA............................................................................................................................................................................................    1     1    JCK-1
.......................cucucccucaaggcuucuaA............................................................................................................................................................................................    1     1    JCK-3
.......................cucucccucaaggcuucuagc...........................................................................................................................................................................................    2     0    JCK-3
........................ucucccucaaggcuucua.............................................................................................................................................................................................    1     0    JCK-1
........................ucucccucaaggcuucua.............................................................................................................................................................................................    2     0    JCK-2
........................ucucccucaaggcuucua.............................................................................................................................................................................................    1     0    JCK-3
........................ucucccucaaggcuucuagc...........................................................................................................................................................................................    1     0    JCK-3
........................ucucccucaaggcuucuagc...........................................................................................................................................................................................    4     0    LCK-3
........................ucucccucaaggcuucuagcg..........................................................................................................................................................................................    3     0    JCK-1
........................ucucccucaaggcuucuagcg..........................................................................................................................................................................................    1     0    JCK-3
........................ucucccucaaggcuucuagcU..........................................................................................................................................................................................    1     1    JCK-3
........................ucucccucaaggcuucuagcg..........................................................................................................................................................................................    3     0    JCK-2
...........................cccucaaggcuucuagcguu........................................................................................................................................................................................    1     0    LT-3
............................................................cuggaagccuuuuggggaga.......................................................................................................................................................................    1     0    LT-1
..............................................................ggaagccuuuuggggagag......................................................................................................................................................................    2     0    LCK-3
..............................................................ggaagccuuuuggggagag......................................................................................................................................................................    1     0    JCK-3
..............................................................ggaagccuuuuggggagag......................................................................................................................................................................    1     0    LT-3
..............................................................ggaagccuuuuggggagagu.....................................................................................................................................................................    1     0    JCK-2
..............................................................ggaagccuuuuggggagagu.....................................................................................................................................................................    2     0    LT-1
..............................................................ggaagccuuuuggggagagu.....................................................................................................................................................................    3     0    LT-3
..............................................................ggaagccuuuuggggagagu.....................................................................................................................................................................    39    0    LCK-3
..............................................................ggaagccuuuuggggagagu.....................................................................................................................................................................    15    0    LT-2
..............................................................ggaagccuuuuggggagaguu....................................................................................................................................................................    21    0    JCK-3
..............................................................ggaagccuuuuggggagaguu....................................................................................................................................................................    23    0    LT-2
..............................................................ggCagccuuuuggggagaguu....................................................................................................................................................................    1     1    LT-1
..............................................................ggaagccuuuuggggagaguu....................................................................................................................................................................    3     0    JT-2
..............................................................ggCagccuuuuggggagaguu....................................................................................................................................................................    1     1    LT-2
..............................................................ggaagccuuuuggggagaguu....................................................................................................................................................................    16    0    LT-1
..............................................................ggaagccuuuuggggagaguu....................................................................................................................................................................    4     0    JT-3
..............................................................ggaagccuuuuggggagaguu....................................................................................................................................................................    13    0    JCK-1
..............................................................ggaagccuuuuggggagaguu....................................................................................................................................................................    28    0    LCK-3
..............................................................ggaagccuuuuggggagaguu....................................................................................................................................................................    19    0    LT-3
..............................................................ggaagccuuuuggggagaguu....................................................................................................................................................................    29    0    JCK-2
..............................................................ggaagccuuuuggggagaguug...................................................................................................................................................................    8     0    JCK-1
..............................................................ggaagccuuuuggggagaguug...................................................................................................................................................................    7     0    LT-1
..............................................................ggaagccuuuuggggagaguuU...................................................................................................................................................................    1     1    JCK-2
..............................................................ggaagccuuuuggggagaguuU...................................................................................................................................................................    1     1    JCK-1
..............................................................ggCagccuuuuggggagaguug...................................................................................................................................................................    1     1    JT-2
..............................................................ggaagccuuuugUggagaguug...................................................................................................................................................................    1     1    JCK-2
..............................................................ggaagccuuuuggggagaguug...................................................................................................................................................................    4     0    JT-3
..............................................................ggaagccuuuuggggagaguug...................................................................................................................................................................    22    0    JCK-2
..............................................................ggaagccuuuuggggagaguug...................................................................................................................................................................    3     0    LT-2
..............................................................ggaagccuuuuggggagaguug...................................................................................................................................................................    12    0    JCK-3
..............................................................ggaagccuuuuggggagaguug...................................................................................................................................................................    2     0    JT-2
..............................................................ggaagccuuuuggggagaguug...................................................................................................................................................................    2     0    LT-3
..............................................................ggaagccuuuuggggagaguug...................................................................................................................................................................    17    0    LCK-3
..............................................................ggaagccuuuuggggagaguuga..................................................................................................................................................................    1     0    JCK-2
..............................................................ggaagccuuuuggggagaguuUa..................................................................................................................................................................    5     1    LCK-3
```

```
                    Mature                               Star
uuauggugacaauuguuuguucacucucccucaaggcuucuagcguugcguacgcaaaagcuggaagccuuuuggggagaguugacagauuguugucacaccuuuuugcaguuuucuacuccuuggacuaguaauauuauuuuauauguaauaucacugcuuuacuuugccuacuugcacucucucuuaucauguuuccuaguuuccauuuuucugaaauuuuguugcaauuugugcugauuaugcuuac
..............................................................ggaagccuuuuggggagaguuga.....................................................................................................................................................................	1	0	JCK-1
...............................................................gaagccuuuuggggagagu........................................................................................................................................................................	4	0	JT-2
...............................................................gaagccuuuuggggagagu........................................................................................................................................................................	1	0	JT-3
```
